# Involvement of mesosalpinx in endometrioma is a possible risk factor for decrease of ovarian reserve after cystectomy: a retrospective cohort study

**DOI:** 10.1186/s12958-016-0210-9

**Published:** 2016-10-28

**Authors:** Ai Saito, Akira Iwase, Tomoko Nakamura, Satoko Osuka, Bayasula, Tomohiko Murase, Nao Kato, Chiharu Ishida, Sachiko Takikawa, Maki Goto, Fumitaka Kikkawa

**Affiliations:** 1 Department of Obstetrics and Gynecology, Nagoya University Graduate School of Medicine, 65 Tsurumai-cho, Showa-ku, Nagoya, 466-8550 Japan; 2 Department of Maternal and Perinatal Medicine, Nagoya University Hospital, 65 Tsurumai-cho, Showa-ku, Nagoya, 466-8550 Japan

**Keywords:** Anti-Müllerian hormone, Cystectomy, Endometriomas, Mesosalpinx, Ovarian reserve

## Abstract

**Background:**

Serum anti-Müllerian hormone (AMH) concentration has been used to assess ovarian reserve in patients with endometriosis, especially when endometrioma surgery is involved. Previously, we reported that decreased serum AMH levels after cystectomy for endometriomas can recover to preoperative levels in some cases. In this present study, we assessed the sequential changes in serum AMH levels before and after cystectomy in terms of the state of the mesosalpinx prior to surgery.

**Methods:**

The retrospective cohort study recruited 53 patients from a series of prospective studies conducted from 2009 to 2015. All patients underwent laparoscopic cystectomy for endometriomas. If either mesosalpinx was involved in the endometrioma or adnexal adhesion before cystectomy, the case was defined as ‘involved mesosalpinx’ (*n* = 14). If both mesosalpinx remained anatomically correct, the case was classified as ‘intact mesosalpinx’ (*n* = 39). Blood samples were obtained from the patients 2 weeks before surgery, and at 1 month and 1 year after surgery to assess serum AMH levels.

**Results:**

The serum AMH levels (the involved group *vs.* the intact group) were 1.92 *vs.* 0.98 (*P* = 0.552) preoperatively, 0.59 *vs.* 1.99 (*P* = 0.049) at 1 month postoperatively, and 0.48 *vs.* 2.37 ng/mL (*P* = 0.007) at 1 year postoperatively. The involved mesosalpinx group showed a further decrease in serum AMH levels at 1 year postoperatively, while serum AMH levels in the intact mesosalpinx group tended to recover.

**Conclusion:**

These results suggest that pre-existing mesosalpinx disturbance, in combination with adhesiolysis, may be involved in the medium- and long-term decrease in ovarian reserve after endometrioma surgery. A disturbance in ovarian blood supply via the mesosalpinx may underlie this.

**Trial registration:**

UMIN-CTR UMIN000019369. Retrospectively registered October 15, 2015.

## Background

Endometriosis, which is frequently seen in women of reproductive age, is one of the most important causes of infertility [1, 2]. Ovarian reserve is an indicator of ovarian potential reflecting the number and quality of remaining follicles [3]. It has been evaluated in association with endometriosis and treatments for this condition [4–10]. Over the past decade, the serum anti-Müllerian hormone (AMH) level has been widely used as a reliable quantitative marker of ovarian reserve [11–14]. Since serum AMH level is not dependent on the phases of the menstrual cycle [15], measurement of serum AMH levels has become part of routine clinical practice even outside of infertility treatments. For example, it has been used to evaluate ovarian damage resulting from surgery or chemotherapy [16, 17].

Laparoscopic cystectomy is the gold standard for managing endometriomas. Evidence suggests that it results in a lower recurrence rate and higher spontaneous pregnancy rate than other types of treatment such as drainage and ablation [18]. However, several studies have demonstrated the decrease of ovarian reserve in terms of serum AMH levels after laparoscopic cystectomy. The accidental loss of ovarian cortex and the damage caused by electrocautery have been proposed as possible causes for this decrease [8, 19, 20].

There is consensus that serum AMH levels decline immediately after cystectomy for endometriomas [19]. However, the medium- and long-term effects of cystectomy on ovarian reserve are still controversial. We have previously reported that the serum AMH levels at 1 year after cystectomy can recover or decline further compared to the AMH levels at 1 month after surgery [20]. Serum AMH levels may recover if the environment surrounding the ovary favors the growing follicle cohort, while less favorable conditions may cause a further decline in ovarian reserve. We speculate that the disturbance of blood supply to the ovary is one such unfavorable condition.

The blood supply to the ovary is supported by the ovarian ligament, suspensory ligament, and mesosalpinx. Advanced endometriosis often involves these structures and causes serious adhesions. One of the main aims of surgery for endometriosis is to improve fertility by restoring the normal anatomy of the reproductive organs; this may be partly achieved by adhesiolysis. However, such procedures risk affecting the blood supply to the ovary.

In this present study, we evaluated the anatomical condition of the mesosalpinx in laparoscopic surgery for endometriomas, and analyzed its relation to ovarian reserve in terms of pre- and post-surgical serum AMH levels.

## Methods

### Patients

A series of prospective studies investigating serum AMH levels in patients with endometriosis were conducted from October 2009 to January 2015 in the Department of Obstetrics and Gynecology of Nagoya University Hospital in Nagoya, Japan. These studies were approved by the ethical committee of Nagoya University Hospital, and informed consent had been obtained from all patients prior to enrollment.

We recruited 53 patients as a retrospective cohort for the current study. The inclusion criteria were as previously described [21]: 1) uni/bilateral endometrioma(s), 2) women aged 20 to 42 years with regular menstrual cycles, and 3) no evidence of any other endocrine disorders. The exclusion criteria were as follows: 1) previous history of adnexal surgery and 2) any findings suspicious of malignant ovarian disease.

### Surgery

All patients underwent laparoscopic surgery under general anesthesia as previously described [21]. Adhesiolysis was performed prior to ovarian cystectomy. The capsules of the endometriomas were stripped from the healthy surrounding ovarian tissue after the cleavage plane had been identified. The use of bipolar forceps to achieve hemostasis was minimized to avoid damage to normal tissue. Sutures were used to close the ovarian parenchyma. Endometriosis was classified according to the revised American Society of Reproductive Medicine (rASRM) classification system [22], and the diagnosis was confirmed by histological examination of the excised specimens.

### Evaluation and classification of mesosalpinx condition

**Fig. 1:**
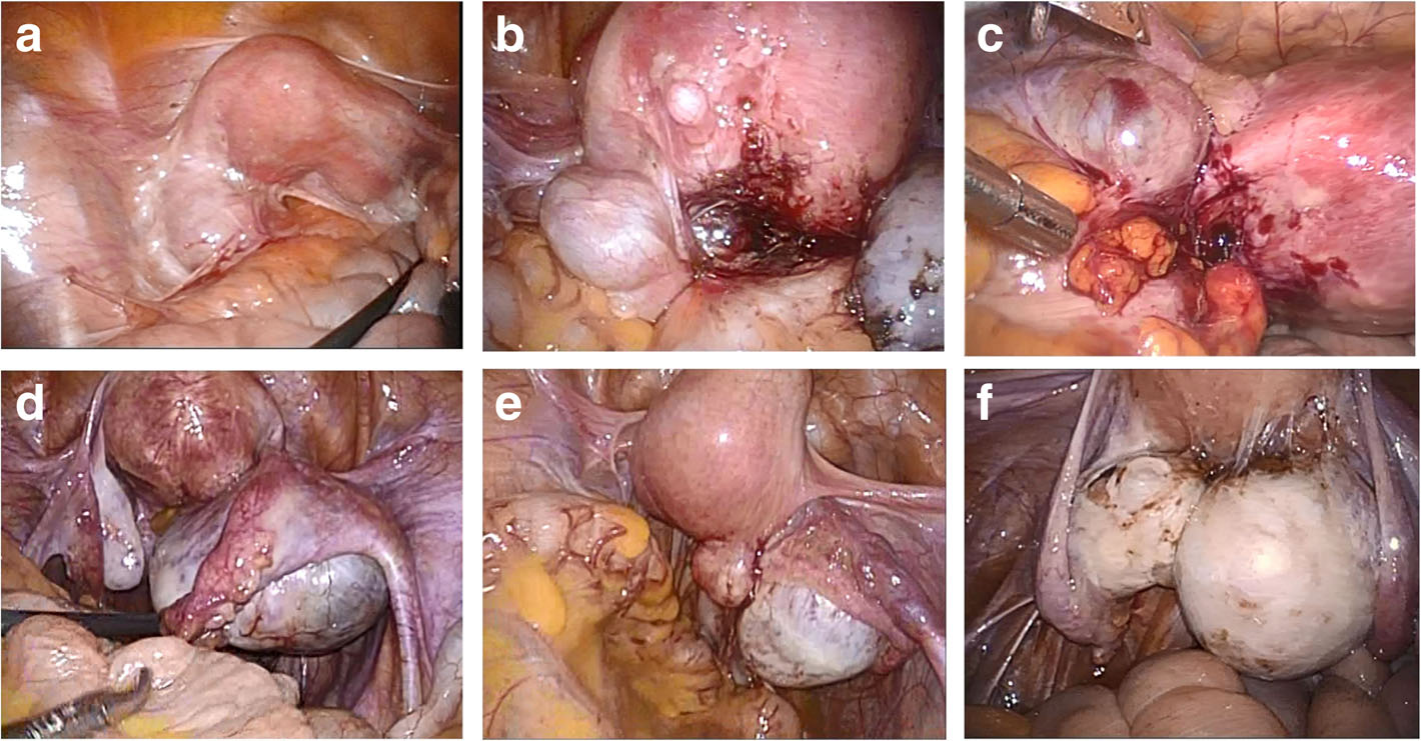
Involved mesosalpinx (**a**–**c**) and intact mesosalpinx (**d**–**f**) in endometrioma

The video recording of each surgery was thoroughly inspected and analyzed by the same investigator (A.I.), who was blinded to the patients’ identities. The investigator focused on the condition of the mesosalpinges before surgery. If the mesosalpinx was affected by the endometrioma or involved in adnexal adhesion, the case was part of the ‘involved mesosalpinx’ group. If both mesosalpinges remained anatomically correct before and after surgery regardless of the presence of endometriomas, the case was classified into the ‘intact mesosalpinx’ group. Representative images of both groups are shown in Fig. 1.

### AMH measurements

Blood samples were obtained from the patients 2 weeks before surgery, and at 1 month and 1 year after surgery. The serum was separated and stored as previously described [20, 23]. The serum AMH concentrations were measured with an enzyme immunoassay kit, AMH GenII (Beckman Coulter, Inc., Brea, CA, USA). The intra-assay and inter-assay variation coefficients were 5.4 % and 5.6 % respectively. Samples with assay values under the AMH Gen II detection limit were assayed using the picoAMH (Ansh Labs, Webster, TX, USA) without dilution by the same operator as described previously [23].

### Histologic analysis

To assess the removal of ovarian tissue, the pathological slides were sliced out of paraffin blocks and selected at equal distances. We counted the number of follicles, including primordial, primary, secondary, and Graafian follicles, from 4 or 5 slides selected according to the size of the cyst wall. This was done using an optical microscope (BX60; Olympus Corporation, Tokyo, Japan) as previously described [20].

### Antral follicle count

Antral follicle count (AFC) was measured by transvaginal ultrasound (5-7.5 MHz transducer) 1 year after surgery. AFC was defined as the total number of follicles with a diameter less than 9 mm [24]. All ultrasound examinations were performed by the same investigator.

### Statistical analysis

All data were analyzed using the SigmaPlot13 software program (Systat Software Inc., San Jose, CA). The student’s *t*-test, Mann-Whitney *U*-test, and Fisher’s exact test were used to compare the patient characteristics and variables of interest between the intact mesosalpinx and involved mesosalpinx groups. *P* < .05 was considered statistically significant.

## Results

A total of 53 patients were recruited, of whom 32 had unilateral endometriomas and 21 had bilateral endometriomas. They were classified into 2 groups, the ‘involved mesosalpinx’ group and the ‘intact mesosalpinx’ group, according to the condition of their mesosalpinges as defined in the materials and methods section. Table 1 shows their clinical characteristics, including data on the operation and the serum AMH levels. There were no statistically significant differences in age, cyst size, blood loss during surgery, number of follicles excised in specimens or preoperative serum AMH levels between the groups. However, there were significant differences in the ratio of unilateral/bilateral cases and the rASRM score between the groups. In addition, the serum AMH levels at 1 month and 1 year after surgery were significantly lower in the involved group compared to those in the intact group.

**Table 1:** Patient characteristics

Characteristics and Variables	Overall (*n* = 53)	Involved mesosalpinx (*n* = 14)	Intact mesosalpinx (*n* = 39)	*P* value
Age [years]	34.1 ± 4.6	34.5 ± 5.1	34.0 ± 4.4	0.711^a^
Preoperative factors				
Cyst size 1 [cm]	6.1 ± 2.0	5.5 ± 1.8	6.3 ± 2.1	0.203^b^
Cyst size 2 [cm]	3.3 ± 1.3	3.4 ± 1.0	3.2 ± 1.6	0.692^a^
Serum CA125 [IU/mL]	54.6 [30.4, 80.9]	49.6 [20.0, 79.8]	54.8 [32.6, 85.1]	0.246^b^
Surgery				
Unilateral/Bilateral [*n* (%)]	32 (60) / 21 (40)	4 (29) / 10 (71)	28 (72) / 11 (28)	0.009^c^
Blood loss [mL]	55 [10, 260]	108 [8.8, 331]	50 [10, 159]	0.327^b^
rASRM score	36 [24, 75]	62 [39, 102]	28 [22, 64]	0.005^b^
Number of follicles in specimens	2.0 [0.0, 6.5]	2.0 [0.0, 6.3]	2.0 [0.0, 8.0]	0.992^b^
Serum AMH [ng/mL]				
Preoperative	2.73 [1.49, 5.57]	1.92 [0.50, 6.92]	2.98 [1.63, 5.27]	0.552^b^
Postoperative 1 month	1.53 [0.49, 3.19]	0.59 [0.15, 2.40]	1.99 [0.75, 3.20]	0.049^b^
Postoperative 1 year	1.76 [0.47, 3.45]	0.48 [0.23, 2.10]	2.37 [0.76, 3.98]	0.007^b^

*Note*: Cyst size 1 represents the mean diameter of the unilateral cyst or the larger cyst in patients with bilateral cysts. Cyst size 2 represents the mean diameter of the smaller cyst in patients with bilateral cysts. The values are presented as the mean ± SD or median [25^th^, 75^th^ percentile]. *P* values in the unilateral *vs*. bilateral groups. ^a^,Student’s *t-*test. ^b^,Mann-Whitney *U* test. ^c^,Fisher's exact test

**Fig. 2:**
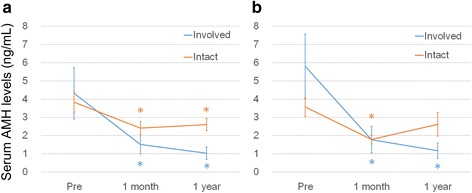
The serial changes in the AMH levels before surgery, 1 month after surgery and 1 year after surgery in all cases (**a**) and in the bilateral cases (**b**)

Figure 2 shows the sequential changes in the serum AMH levels. Both groups had significant decreases in the serum AMH levels at 1 month and 1 year after surgery compared to the preoperative levels. However, the serum AMH levels were unchanged at 1 year after surgery in the intact group (Fig. 2a). The bilateral cases in the intact group showed a slight recovery in serum AMH level at 1 year after surgery, while the bilateral cases in the involved group showed a greater decrease at 1 year compared to that at 1 month after surgery (Fig. 2b).

We then compared the AFC in the unilateral cases of both groups. In the involved mesosalpinx group, we found a large decrease in the AFC of the affected ovary than in that of the control ovary. In the intact mesosalpinx group, the difference in AFC between the affected ovary and control ovary was much smaller, albeit still statistically significant (Table 2).

**Table 2:** Postoperative antral follicle counts in the unilateral cystectomy group

	Affected ovary	Control ovary	*P* value
Involved mesosalpinx (*n* = 4)	1.0 ± 1.2	2.3 ± 1.1	0.015^a^
Intact mesosalpinx (*n* = 28)	2.8 ± 1.6	3.6 ± 2.0	<0.001^a^

*Note*: The values are presented as the mean ± SD. *P* values in the affected ovary *vs*. control ovary. ^a^,Paired *t-*test

## Discussion

In the current study, we demonstrated that mesosalpinx disturbance, in tandem with adhesiolysis, has a larger impact on the decline of AMH levels at 1 year after surgery than at 1 month after surgery. Our results suggest that serum AMH levels can recover if the follicle cohort is restored, and that an adequate ovarian blood supply is needed for this to take place.

Several clinical studies evaluating serum AMH levels have suggested the importance of the ovarian blood supply in determining ovarian reserve [25, 26]. Studies analyzing AMH levels in salpingectomy cases have provided useful information with which our results can be discussed. Cross-sectional studies have reported controversial results: 2 studies reported a decrease in serum AMH levels after salpingectomy compared to that in cases that had not undergone tubal surgery [27, 28]; on the contrary, Ni *et al*. reported that tubal surgery improved the outcome of in vitro fertilization without negatively impacting serum AMH levels [29]. To our best of our knowledge, only 3 reports comparing pre- and post-surgical AMH levels in fallopian tube surgery have been published. Studies on tubal ligation or tubal dissection did not find a significant decrease in AMH levels [30, 31]. Venturella *et al.* reported a decline in AMH levels after salpingectomy, although they noted that the decrease of AMH levels between the usual salpingectomy and “wide salpingectomy” with removal of mesosalpinx groups were not significantly different [32]. However, the average age of the study participants exceeded 40 years, and the average preoperative AMH levels were less than 1.0 ng/mL. This means that their results are less applicable to females in their prime childbearing years, as in our study.

There is a consensus that serum AMH levels decline immediately after cystectomy for endometriomas [19]. However, studies on the medium- and long-term effects of cystectomy on ovarian reserve show conflicting results. Celik *et al.* assessed serum AMH levels preoperatively and at 6 weeks and 6 months postoperatively. They reported that AMH levels gradually decreased in patients with bilateral tumors or those with cyst diameters ≥5 cm [5]. Ozaki *et al.* also reported that the reduced serum AMH concentrations at 6 months after surgery were similar to those at 3 months after surgery [33]. In our previous study, we showed that both cases, increase and decrease of AMH levels at one year after cystectomy, are comparative. On the other hand, 2 studies reported that serum AMH levels at 6 or 12 months after surgery had recovered to preoperative levels [34, 35]. This difference might be explained by the preservation of the ovarian blood supply. In cases of advanced endometriosis with severe adhesions, recovery of serum AMH levels might prove difficult because of the damage to the mesosalpinx caused by adhesiolysis.

Recently, several clinical studies measuring serum AMH levels preoperatively and postoperatively have been conducted to evaluate the methods used to achieve hemostasis. Most of these studies compared suturing and bipolar coagulation. Excessive bipolar coagulation causes damage to the ovarian cortex, resulting in a postoperative decline in ovarian reserve [36]. On the other hand, Shao *et al.* found a postoperative decline in serum AMH levels in the suturing group after cystectomy [37]. The sutures might have been too tight, which could have interrupted the ovarian blood supply. This should be avoided in order to maintain ovarian reserve.

One limitation of our study was that the blood supply to ovary was not assessed directly. The ovarian blood supply is mediated by the ovarian ligaments, suspensory ligament and mesosalpinx, and is extremely complex. Color Doppler ultrasonography is useful for evaluating blood flow in a wide range of organs and tissues; however, an optimal method for assessing ovarian blood supply quantitatively has yet to be established. Further studies should explore this. This may help to improve our understanding of the correlation between the postoperative changes in ovarian blood supply and the decline of ovarian reserve.

We have previously reported that compared to pregnant women, non-pregnant women had lower serum AMH levels 1 year after cystectomy for endometriomas [21]. This suggested that a decrease in serum AMH levels 1 year after cystectomy might negatively impact future fertility. As such, we need to reconsider our surgical approach to endometriosis, such that the ovarian blood supply – mediated by adnexal structures, especially the mesosalpinx – is maintained.

## Conclusions

In conclusion, we demonstrated that the involvement of mesosalpinx in endometrioma is a possible risk factor for decrease of ovarian reserve after cystectomy. The mesosalpinx disturbance, in tandem with adhesiolysis, may have a negative impact on the ovarian blood supply. Our results suggest that an adequate ovarian blood supply is required for the restoration of the follicle cohort followed by recovery of serum AMH levels.

## Abbreviations

AFC: Antral follicle count
AMH: Anti-Müllerian hormone
rASRM: Revised American society of reproductive medicine.
